# The relationship between the cranial base and jaw base in a Chinese population

**DOI:** 10.1186/1746-160X-10-31

**Published:** 2014-08-16

**Authors:** Alice Chin, Suzanne Perry, Chongshan Liao, Yanqi Yang

**Affiliations:** 1 Hong Kong SAR, China; 2 Faculty of Dentistry, University of Hong Kong, Hong Kong SAR, China

**Keywords:** Cranial base, Jaw base, Chinese

## Abstract

**Introduction:**

The cranial base plays an important role in determining how the mandible and maxilla relate to each other. This study assessed the relationship between the cranial base and jaw base in a Chinese population.

**Methods:**

This study involved 83 subjects (male: 27; female: 56; age: 18.4 ± 4.2 SD years) from Hong Kong, who were classified into 3 sagittal discrepancy groups on the basis of their ANB angle. A cephalometric analysis of the angular and linear measurements of their cranial and jaw bases was carried out. The morphological characteristics of the cranial and jaw bases in the three groups were compared and assessments were made as to whether a relationship existed between the cranial base and the jaw base discrepancy.

**Results:**

Significant differences were found in the cranial base angles of the three groups. Skeletal Class II cases presented with a larger NSBa, whereas skeletal Class III cases presented with a smaller NSBa (P *<* 0.001). In the linear measurement, skeletal Class III cases presented with a shorter NBa than skeletal Class I and II cases (P < 0.01). There was a correlation between the cranial base angle NSBa and the SNB for the whole sample, (r = -0.523, P < 0.001). Furthermore, correlations between SBaFH and Wits (r = -0.594, P < 0.001) and SBaFH and maxillary length (r = -0.616, P < 0.001) were more obvious in the skeletal Class III cases.

**Conclusions:**

The cranial base appears to have a certain correlation with the jaw base relationship in a southern Chinese population. The correlation between cranial base and jaw base tends to be closer in skeletal Class III cases.

## Introduction

The cranial base separates the delicate tissues of the brain from the rest of the face and has a major influence on growth. The main postnatal growth site is the spheno-occipital synchondrosis, which lengthens the base of the skull. The positioning of the maxilla anterior to the synchondrosis and the mandible, which articulates posteriorly, gives the synchondrosis the potential to be a factor in facial disharmony and consequently malocclusion. The synchondrosis influences growth in the region until shortly after puberty, when it fuses. Growth at the spheno-occipital synchondrosis increases the length of the cranial base, and as the maxillary complex lies beneath the anterior cranial fossa and the mandible articulates with the skull at the temporomandibular joint, which lies beneath the middle cranial fossa, the cranial base plays an important part in determining how the mandible and maxilla relate to each other. The cranial base can be split into two parts, the anterior and the posterior; the anterior is measured from the foramen caecum to the sella turcica (S) and the posterior from the sella turcica to the basion (Ba). The growth of the cranial base in very early years follows a neural pattern, with the most rapid rate of growth in the first 3 years [1]. As a result, variations in the cranial base angle and the anterior and posterior lengths can potentially be a cause of imbalances in facial growth, and consequently occlusion.

Moss’ functional matrix theory believes that environmental demands mold the pattern of growth of the genetically predetermined facial bones to result in a final outcome [2]. Van Limborgh’s Compromise Theory accepts functional matrix theory of Moss but also supports some aspects of Sutural theory of Sicher and acknowledges genetic involvement [3]. The growth of the cranial base appears to be more genetically controlled than environmentally affected and it is the cranial base synchondroses that are the major growth centers of the cranial base [4]. Genetics appear to play such a big role in facial growth that genetic influence is more apparent in some malocclusions than others, as can be seen in skeletal Class III cases [5]. So how much influence does the cranial base have on the jaw relationship?

Some studies have linked a reduction in cranial base angle, that is a more acute angle, to a more relatively anterior articulation of the eminence of the mandible with the glenoid fossa, which is more likely to lead to a Class III type malocclusion [6]; however, a more obtuse cranial base angle may be a causative factor in a Class II situation [7-9]. Anderson and Popovich, in a serial sample of data from the Burlington Growth Centre, and others, have observed an increasing cranial base angle spanning Class III to Class I to Class II malocclusions [10-14]. However, some debate has arisen due to conflicting results from other studies, and the matter remains inconclusive. An interesting study by Rothstein and Phan looked at 335 boys and girls between 10 and 14 with Class II Division 1 malocclusions and compared them cephalometrically to controls; they found no correlation between cranial base angle and sagittal jaw relationship between the two groups [15]. A similar study by Wilhelm et al. comparing Class I subjects to Class II subjects found no relationship between the cranial base angle and the skeletal growth pattern [16]. Dhopatkhar et al. were also unable to find any influence of the cranial base angle in four types of malocclusion [17].

Given these inconclusive findings, we would like to raise one other issue—racial differences. Scientific awareness of variations in racial cranial morphology has existed since the mid-1700’s, when the German scientist Johan F. Blumenbach described the different features of skulls from five world regions. He commented on the different characteristics of skulls and hypothesized that the variations were due to factors such as diet, geographical location, and even specific mannerisms [18]. Other studies have also noted variations in cranial base angles in different races and notable differences in general craniofacial forms between races [19,20]. Furthermore, the prevalence of intermaxillary jaw discrepancy also varies between races. Angle first described such jaw relationships in 1890. Using the molar relationship, he classified malocclusion in his sample of Caucasians; he found that 69% had a normal or Class I occlusion, 24% had a Class II and around 3% had a Class III malocclusion (other 4% were unclassified due to teeth missing) [21]. As the majority of studies have been carried out in Caucasian populations, it is unclear how much influence the cranial base has on the jaw base relationship in a Chinese population. The importance of acquiring data relevant to a particular subgroup cannot be underestimated. To avoid the heterology of China, we focus on a southern Chinese population. The aims of this investigation are to assess if there is any evidence that the cranial base angle predisposes the jaw base relationship in a southern Chinese population, and to gain data specific to a southern Chinese population with particular reference to the angular and linear cranial base morphology.

## Materials and methods

### Samples

The samples for this retrospective study which was ethically approved were obtained from patients attending the orthodontic clinic at the Faculty of Dentistry, the University of Hong Kong during the period of 2012–2013 in a consecutive series. Their consent to use their clinical records for research purposes was obtained. All of the subjects were Hong Kong residents, of southern Chinese origin, and healthy with no evidence or history of medical complications, craniofacial malformation, or syndromes. Any subject with a previous history of orthodontic treatment was excluded from the study. Subjects with severe crowding were also excluded.

Based on a pilot study that we conducted, variance within groups (skeletal Classes I, II, and III) in cranial base angle (NSBa) was 4.9 degrees. Each group required 26 patients to yield a 95% power for identifying a significant difference in a one-way ANOVA at a 5% level of significance (alpha = 0.05). The power analysis was undertaken by G*Power 3.1.7 (a program developed by Axel Buchner, Edgar Erdfelder, and Franz Faul; http://www.psycho.uni-duesseldorf.de/abteilungen/aap/gpower3).

The final sample was comprised of 83 patients (male: 27; female: 56; age: 18.4 ± 4.2 SD years).

### Cephalometric analysis

Lateral cephalometric radiographs were taken in centric relation as part of a routine orthodontic diagnostic process using a GE1000 (General Electric, Milwaukee, Wisconsin) machine with subjects in a natural head posture position. It was estimated that the magnification for a mid-sagittal structure would be close to the value of 8.8%. Subjects were then allocated into three defined groups of Class I, Class II, and Class III on the basis of their ANB angulations with the Chinese norm as the reference [22]. The criteria for the three classes were as follows:

*skeletal class I*, *ANB angle of 0.6°- 5° with a favorable overjet and overbite;*

*skeletal class II*, *ANB angle of ≥5° with an increased overjet; and*

*skeletal class III*, *ANB angle of < 0.6° with a reduced overjet.*

**Figure 1:**
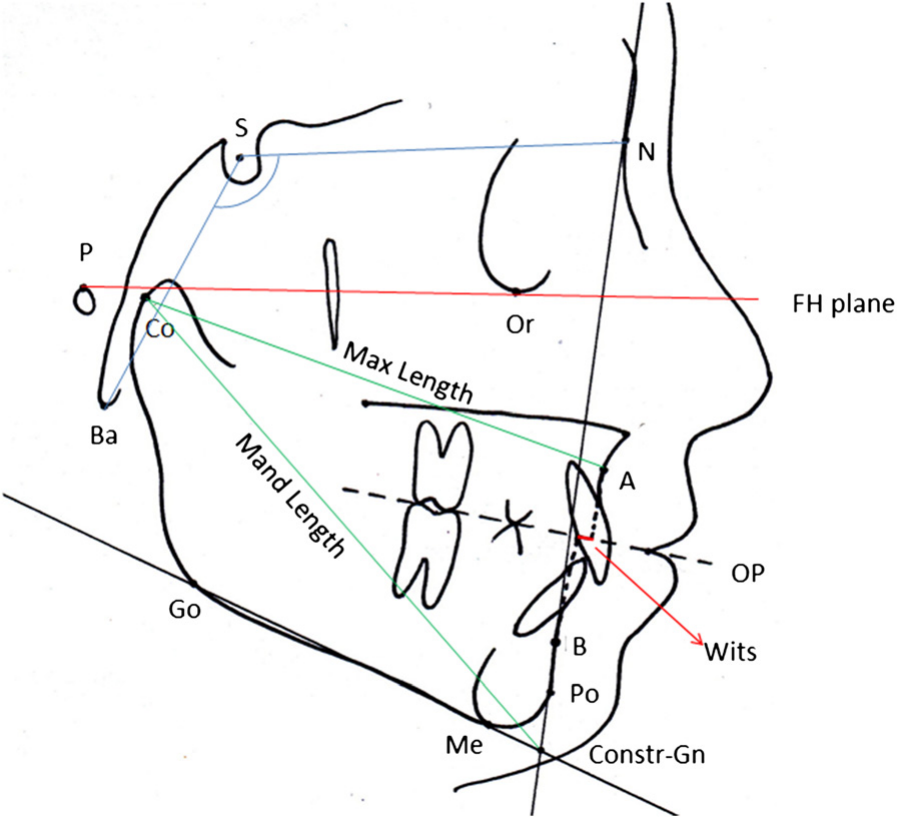
**The cephalometric landmarks used in this study.** Reference point: cranial base: N (Nasion), S (Sella), P (Porion), Ba (Basion), Or (Orbitale); jaw base: Co (Condylion), Go (Gonion), Me (Menton), A (Supramentale), B (Supramentale), Po (Pogonion). Reference plane: FH (Frankfort plane), OP (Occlusal plane), Go-Me (Mandibular plane).

The radiographs were first traced by hand in a darkened room by a trained and calibrated orthodontist and then digitized (CASSOS 2001, Soft Enable Technology Limited, Hong Kong). The average value was taken of any double features not present on the mid-sagittal plane. A cephalometric analysis of the cranial base including angular measurements (NSBa and SBaFH) and linear measurements (SN, SBa, NBa, Wits) was carried out [23]. Jaw base length and relationship was assessed in the sagittal and vertical dimensions. The cephalometric variables analyzed in this study are shown in Figure 1.

### Statistical analysis

For each of the three morphological subtypes, the means and standard deviations were calculated for each cephalometric variable in each group. A One-way Analysis of Variance (ANOVA) was carried out to compare the characteristics of cranial bases and jaw bases between the three groups. A Pearson Correlation Coefficient was calculated between each cephalometric variable with particular emphasis on the relationships between the cranial base angle and the sagittal jaw discrepancy markers for the whole sample and the three groups. Significance for the tests was noted at three levels, P < 0.05 (*), P < 0.01 (**), and P < 0.001 (***). The correlation was regarded as meaningful when r > =0.5 in addition to the significance revealed by P < 0.05, whereas the correlation was regarded as weak when r < 0.5, even if there was some statistical significance (P < 0.05). All of the statistical analyses were performed using Statistical Package for Social Sciences software package (SPSS for Windows, Version 10.0, Chicago, IL, USA).

## Results

### Cephalometric profile of the cranial base and jaw base of a southern Chinese sample

There were 27 subjects in the Class I group (18.1 ± 3.3 years old), 30 in the Class II group (19.1 ± 5.6 years old), and 26 in the Class III group (18 ± 3.2 years old). No significant difference was shown in the ages of the three groups (P > 0.05).

The cephalometric values for the whole sample and for each subgroup are presented in Table 1. For the cranial base, the angular measurement showed that there was a significant difference in the NSBa angle between the three groups (P < 0.001): the Class II group had a larger cranial base angle (NSBa) (131.9 ± 5.2), whereas the Class III cases had a smaller NSBa (127.0 ± 3.7). In the linear measurement, the Class III cases had a shorter NBa (105.0 ± 5.4) than the Class I (108.4 ± 5.8) and Class II cases (109.7 ± 4.0), (P < 0.01). For the jaw base relationship, the differences in the sagittal discrepancies among the three groups can be seen by the variation in SNB, ANB, Wits, maxillary length, and the mandibular length, all of which showed significance at P < 0.001. Vertically, the Class III cases had a lower maxillary-mandibular plane angle (MMPA) (24.1 ± 2.4) compared to the Class I (27.0 ± 2.3) (P < 0.001) and Class II cases (26.4 ± 2.4) (P < 0.01).

**Table 1:** Cephalometric profile of the cranial base and jaw base in a Southern Chinese sample

			**Class I (n=27)**		**Class II (n=30)**		**Class III (n=26)**		**Total (n=83)**		**P value (ANOVA)**	
			**Mean**	**SD**	**Mean**	**SD**	**Mean**	**SD**	**Mean**	**SD**		
Cranial base	Angular measurements (^o^)	NSBa	130.0	4.4	131.9	5.2	127.0	3.7	130.0	4.9	0.001	***
		SBaFH	58.7	4.6	57.1	4.1	59.2	3.3	58.2	4.1	0.125	ns
	Linear measurements (mm)	SN	70.1	4.4	70.4	2.8	68.7	3.3	69.8	3.6	0.177	ns
		SBa	49.5	4.4	49.9	3.4	48.2	3.3	49.3	3.8	0.216	ns
		NBa	108.4	5.8	109.7	4.0	105.0	5.4	107.8	5.4	0.003	**
Jaw base	Angular measurements (^o^)	SNA	81.1	3.0	82.1	3.1	82.1	3.9	81.8	3.3	0.474	ns
		SNB	78.3	3.1	75.9	3.0	84.3	5.2	79.3	5.2	0.000	***
		ANB	2.8	1.2	6.2	1.3	-2.4	3.0	2.4	4.0	0.000	***
		MMPA	27.0	2.3	26.4	2.4	24.1	2.4	25.9	2.6	0.000	***
	Linear measurement (mm)	Wits	-3.5	3.5	1.6	2.6	-7.6	8.3	-2.9	6.4	0.000	***
		Max length	87.9	4.6	89.7	3.8	85.6	4.1	87.8	4.5	0.002	**
		M and length	122.0	7.1	117.4	7.0	126.4	9.0	121.7	8.4	0.000	***

*P < 0.05; **P < 0.01; ***P < 0.001; ns (no significance): P > 0.05.

### Correlation between the cranial base measurements

The two angular variables NSBa and SBaFH, which were based on different reference planes, showed significant correlation in the whole sample (r = -0.706; P < 0.001) (Table 2). The correlation between the angular measurement (i.e., NSBa) and linear measurement (i.e., SBa) existed in skeletal Class I cases (r = -0.628, P < 0.001), but not in Class II and III cases. Among the linear variables, it was found that NBa was correlated with both SBa (r = 0.764, P < 0.001) and SN (r = 0.743, P < 0.01), but SBa and SN did not have a strong correlation (r = 0.461, P < 0.001).

**Table 2:** Correlation (r) between the cranial base measurements in a Southern Chinese sample

	**Class I (n=27)**	**Class II (n=30)**	**Class III (n=26)**	**Total (n=83)**
NSBa-SBaFH	-0.692***	-0.898***	-0.332 (ns)	-0.706***
NSBa-NBa	-0.335 (ns)	0.209 (ns)	0.457*	0.211 (ns)
NSBa-SBa	-0.628***	-0.216 (ns)	0.036 (ns)	-0.200 (ns)
NSBa-SN	-0.239 (ns)	-0.177 (ns)	0.248 (ns)	-0.001 (ns)
SBaFH-NBa	0.167 (ns)	-0.297 (ns)	-0.318 (ns)	-0.162 (ns)
SBaFH-SBa	0.290 (ns)	0.119 (ns)	-0.053 (ns)	0.111 (ns)
SBaFH-SN	0.039 (ns)	0.024 (ns)	-0.293 (ns)	-0.081 (ns)
NBa-SBa	0.811***	0.707***	0.754***	0.764***
NBa-SN	0.728***	0.595***	0.851***	0.743**
SBa-SN	0.543**	0.240 (ns)	0.455*	0.461***

*P < 0.05; **P < 0.01; ***P < 0.001; ns (no significance): P > 0.05.

Bolded: r > 0.5.

### Correlation between the cranial base and jaw base

The analysis of the cranial base angle for the whole sample showed a noticeable correlation in the sagittal jaw base between NSBa and SNB (r = 0.523, P < 0.001), indicating that the SNB angle decreases as the cranial base angle increases (Table 3). The correlation of NSBa with ANB and Wits were weak (r < 0.5, P < 0.01), so were the correlation of SBaFH with Wits and maxillary length (r < 0.5, P < 0.001) for the whole sample. However, SBaFH had a stronger correlation with Wits (r = -0.594, P < 0.001) and maxillary length (r = -0.616, P < 0.001) for skeletal Class III cases.

**Table 3:** Correlation (r) between the cranial base angle and jaw base in a Southern Chinese sample

	**Class I (n=27)**	**Class II (n=30)**	**Class III (n=26)**	**Total (n=83)**
NSBa-SNA	-0.423*	-0.449*	-0.372 (ns)	-0.372***
NSBa-SNB	-0.489**	-0.420*	-0.312 (ns)	-0.523***
NSBa-ANB	0.219 (ns)	-0.100 (ns)	0.110 (ns)	0.384***
NSBa-wits	0.238 (ns)	0.031 (ns)	0.006 (ns)	0.280**
NSBa-MMPA	0.160 (ns)	-0.116 (ns)	-0.210 (ns)	0.110 (ns)
NSBa-Max length	-0.235 (ns)	0.001 (ns)	0.349 (ns)	0.161 (ns)
NSBa-Mand length	-0.447*	-0.097 (ns)	0.056 (ns)	-0.362***
SBaFH-SNA	0.116 (ns)	0.276 (ns)	-0.169 (ns)	0.071 (ns)
SBaFH-SNB	0.172 (ns)	0.226 (ns)	-0.236 (ns)	0.159 (ns)
SBaFH-ANB	-0.209 (ns)	0.136 (ns)	0.136 (ns)	-0.164 (ns)
SBaFH-wits	-0.249 (ns)	0.010 (ns)	-0.594***	-0.363***
SBaFH-MMPA	0.005 (ns)	-0.009 (ns)	0.240 (ns)	-0.002 (ns)
SBaFH-Max length	-0.063 (ns)	-0.063 (ns)	-0.616***	-0.262*
SBaFH-Mand length	0.142 (ns)	0.222 (ns)	-0.420*	0.093 (ns)

*P < 0.05; **P < 0.01; ***P < 0.001; ns (no significance): P > 0.05.

Bolded: r >0.5.

For the cranial base length to jaw base relationship (Table 4), none of the linear variables of the cranial base correlated strongly with the sagittal jaw base relationship except for a negative correlation between NBa and SNA in skeletal Class III cases (r = -0.592, P < 0.001). NBa had the same correlated tendency to SNB (r = -0.486, P < 0.05), but not to ANB (P > 0.05) in skeletal Class III cases.

**Table 4:** Correlation (r) between the cranial base length and jaw base relationship in a Southern Chinese sample

	**Class I (n=27)**	**Class II (n=30)**	**Class III (n=26)**	**Total (n=83)**
NBa-SNA	0.007 (ns)	-0.040 (ns)	-0.592***	-0.231*
NBa-SNB	-0.016 (ns)	0.029 (ns)	-0.486*	-0.400***
NBa-ANB	0.063 (ns)	-0.168 (ns)	0.133 (ns)	0.345***
NBa-wits	0.213 (ns)	0.092 (ns)	0.317 (ns)	0.379***
SBa-SNA	0.259 (ns)	0.203 (ns)	-0.476*	-0.006 (ns)
SBa-SNB	0.320 (ns)	0.208 (ns)	-0.296 (ns)	-0.101 (ns)
SBa-ANB	-0.174 (ns)	-0.007 (ns)	-0.088 (ns)	0.132 (ns)
SBa-wits	0.169 (ns)	0.168 (ns)	0.239 (ns)	0.245*
SN-SNA	-0.163 (ns)	-0.044 (ns)	-0.443*	-0.222*
SN-SNB	-0.204 (ns)	-0.020 (ns)	-0.448*	-0.317**
SN-ANB	0.161 (ns)	-0.056 (ns)	0.267 (ns)	0.247*
SN-wits	0.294 (ns)	0.105 (ns)	0.285 (ns)	0.290**

*P < 0.05; **P < 0.01; ***P < 0.001; ns (no significance): P > 0.05.

Bolded: r > 0.5.

There was no correlation between any angular or linear measurement of the cranial base and MMPA, which defines the vertical skeletal pattern (P > 0.05) (Table 5).

**Table 5:** Correlation (r) between the cranial base and the MMPA in a Southern Chinese sample

	**Class I (n=27)**	**Class II (n=30)**	**Class III (n=26)**	**Total (n=83)**
NSBa-MMPA	0.160 (ns)	-0.116 (ns)	-0.210 (ns)	0.110 (ns)
SBaFH-MMPA	0.005 (ns)	-0.009 (ns)	0.240 (ns)	-0.002 (ns)
NBa-MMPA	0.057 (ns)	-0.204 (ns)	-0.258 (ns)	0.050 (ns)
SBa-MMPA	-0.093 (ns)	0.007 (ns)	-0.368 (ns)	-0.037 (ns)
SN-MMPA	0.005 (ns)	-0.085 (ns)	-0.033 (ns)	0.059 (ns)

ns (no significance): P > 0.05.

In the relationship between cranial base length and jaw base length (Table 6), NBa was related to maxillary length in the whole sample (r = 0.665, P < 0.01), but not related to mandibular length in the Class III subgroup (P > 0.05), which means that the shorter NBa is, the shorter the maxillary length is; however, there was no influence on mandibular length in the Class III cases. Similarly, SN was found to be correlated with maxillary length for the whole sample (r = 0.594, P < 0.001), but not mandibular length (P > 0.05). SBa showed low correlation with both maxillary length (r = 0.455, P < 0.001) and mandibular length (r = 0.261, P < 0.05) in the whole sample.

**Table 6:** Correlation (r) between the cranial base length and jaw length in a Southern Chinese sample

	**Class I (n=27)**	**Class II (n=30)**	**Class III (n=26)**	**Total (n=83)**
NBa-Max length	0.685***	0.574***	0.560**	0.665**
NBa-Mand length	0.581***	0.394*	0.243 (ns)	0.173 (ns)
SBa-Max length	0.667***	0.218 (ns)	0.264 (ns)	0.455***
SBa-Mand length	0.703***	0.267 (ns)	0.183 (ns)	0.261*
SN-Max length	0.637***	0.463**	0.580**	0.594***
SN-Mand length	0.366 (ns)	0.320 (ns)	0.193 (ns)	0.165 (ns)

*P < 0.05; **P < 0.01; ***P < 0.001; ns (no significance): P > 0.05.

Bolded: r > 0.5.

## Discussion

### Cranial base in relation to jaw base angle

In the choice of cranial base landmarks, debate has arisen over the use of the Articulare instead of the Basion (Ba) [24], suggesting that it is easier to identify. However, it can be argued that the Ba is closer to the cranial base and is more likely to be valid. Previous studies have shown that the correlation between the two points is high and the choice between them is unlikely to affect a study’s results [25]. Therefore, based on the previous research [26], the Ba was chosen as the landmark point in this study. Previous studies have noted a possible improvement in validity using the Frankfurt (FH) plane in the measurements of the cranial base—they suggest that FH plane has less variation due to a balance in bone remodeling [27]. SBaFH is another variable that measures the cranial base angle using FH as the reference line. Hence, using SBaFH to assess the cranial base angle may reinforce the potential validity of the results.

We found an inverse correlation between the cranial base angle NSBa and the jaw base variable SNB (r = -0.523, P < 0.001) (Table 3), that is, an increased NSBa was accompanied by a reduced SNB, leading to a more Class II profile, and vice versa. This result would seem logical, as the mandible would be positioned more posteriorly on the posterior cranial base leg, coinciding with previous studies [10-14,26]. However, this result was not repeated when the correlation of SBaFH to SNB was analyzed, nor to ANB (Table 3). These results perhaps are due to both NSBa and SNB share the same reference plane SN; but for SNB (with SN as the reference plane) and SBaFH (with FH as the reference plane), the individual variation of the SN-FH angle must be an interference factor.

For the interaction between the SBaFH angle and the linear variables (Table 3), it is helpful to use Wits analysis. The correlation between SBaFH and Wits was shown to be weak in the whole sample (r = -0.363, P < 0.001), but stronger in the skeletal Class III group (r = 0.594, P < 0.001). Similarly, the correlation between SBaFH and maxillary length was also stronger in skeletal Class III cases (r = -0.616, P < 0.001). The negative correlation of SBaFH to Wits and maxillary length indicated that a higher SBaFH was related to a lower Wits and maxillary length, which indicates that the correlation between cranial base and jaw base is closer in skeletal Class III cases than in the other malocclusions. Here we would like to clarify that SBaFH is an acute angle and an increase in SBaFH represented a reduced NSBa, which coincided with a significant correlation between NSBa and SBaFH (r = -0.706, P < 0.001) (Table 2). Hence, these results coincided with the results of NSBa and SNB.

The relationship between the cranial base and the maxilla was first noted by Jarvinen who published the link between SNA and the cranial base: an increased cranial base angle would lead to a smaller SNA [28], and this link was later explained with a detailed statistical analysis [13]. Further studies have shown that the correlation between the two values was probably high due to topographical factors, most likely the rotation of the SN plane [12,17]; thus the SN value was deemed an unreliable indicator. As a result, it has been suggested that the position of the maxilla is likely to be determined more by genetic or epigenetic factors rather than directly by the cranial base [29]. In our study, we did not find any correlation between SBaFH and SNA (P > 0.05) and only a weak correlation between NSBa and SNA (r = -0.372, P < 0.001) (Table 3). The linear variable NBa had a stronger correlation with SNA (r = -0.592, P < 0.001) in skeletal Class III samples (Table 4), possibly because both of the parameters share the same reference point N, which may lead to a closer correlation regardless of the individually varied positions of point N. However, the weak correlation between NBa and SNB (r = -0.486, P < 0.05) made the correlation between NBa and ANB insignificant (P > 0.05). Therefore, there is no obvious evidence supporting the relationship between cranial base and maxillary position.

Vertical discrepancies can affect the sagittal position due to a downward and backward rotation of the mandible. In this study, Class III cases had lower MMPA than Class I and Class II cases (Table 1), which confirmed the correlation in the sagittal and vertical dimensions. Jarvinen looked at the cranial base angle in relation to the vertical facial pattern, and found the low angle group had a larger cranial base angle, and the high angle group had a shorter cranial base [13]. This conflicts with the results of this study, specifically, that Class II cases had a higher MMPA than Class III cases (but not Class I cases) and a larger NSBa, whereas Class III cases had a lower MMPA and a smaller NSBa (Table 1). Furthermore, in this study, we did not find any significant correlation between any angular or linear measurements of the cranial base and MMPA (Table 5). Therefore, we are unable conclude that there is a correlation between the cranial base and the vertical skeletal pattern.

### Cranial base in relation to jaw base length

The skeletal discrepancy can be caused by an abnormal jaw position or insufficient/overgrowth of the jaws, leading to an abnormal maxillary and/or mandibular length. In this study, the maxillary length was taken from the Condylion to Point A and the mandibular length was defined from the Condylion to the constructed Gnathion. The correlation between NSBa and mandibular length was extremely weak (r = -0.362, P < 0.001), but the correlation between SBaFH and maxillary length was stronger in skeletal Class III cases (r = -0.616, P < 0.001) (Table 3). Again, this supported the suggestion that an increased SBaFH (as with a decreased NSBa) was related to a reduced maxillary length, i.e., a Class III problem, which also supported the closer correlation between cranial base and jaw base in skeletal Class III cases.

The correlation between cranial base length and jaw length was also assessed. Geometrically, the length of the posterior cranial base in particular has a significant role to play in the sagittal presentations. Previous studies have suggested that a longer posterior cranial base can exacerbate a sagittal Class II situation and a shorter base may increase the chance of a Class III relationship [6,7,27,30]. In contrast, other studies have not been able to confirm such findings regarding cranial base length, but still report some significance differences in angle [14]. In this study, the posterior cranial base length SBa was only found to be correlated to maxillary length and mandibular length in skeletal Class I cases, not in Class II or Class III cases (Table 6). This correlation to both maxillary and mandibular length showed the same change tendency (positive correlation) for skeletal Class I. The cranial base length NBa was strongly correlated to both maxillary and mandibular length in skeletal Class I cases, but only related to maxillary length in skeletal Class III cases. This suggested that the shorter the cranial base, the shorter the maxillary length, i.e., a Class III problem. These results further proved that the correlation between cranial base and jaw base was more obvious in skeletal Class III cases. The importance of considering the cranial base was also shown in a study by Andria et al. which suggested a shorter posterior cranial base in Class II patients may lead to an increased treatment time [27]. Combined with a more obtuse cranial base angle, a shorter posterior cranial base would lead to a higher level placement of the condyle and glenoid fossa, potentially leading to increased MMPA and a greater vertical component to mandibular growth.

### Factors of age and gender

To hypothesize future changes from current patterns, and so produce effective and successful clinical results, it is important to have a good understanding of a process. The growth of the cranial base in the very early years follows a neural pattern, with the most rapid rate of growth in the first 3 years [1]. The cranial base angle is reasonably stable after the age of five [10,31]. Changes in angular and linear parameters during the observation period occurred mostly between the ages of 10 and 12 years [32]. The synchondrosis influences growth in the region until shortly after puberty when it fuses [4]. After puberty, the angle appears to remain stable [33]. In this study, we chose a sample comprised of young adults (mean age: 18.4 years old) to exclude the interference from unknown growth.

The pilot study revealed a similar correlation tendency among the cephalometric variables in males and females. Therefore, in this study, we analyzed the data for males and females together.

### Racial differences

Craniofacial variation between different races has been well documented, with Africans typically having a more dolicocephalic shape and the Mongoloids a more brachycephalic shape than Caucasians [20]. A Finnish study related historical skulls to present-day populations, and showed the differences in inter-racial craniofacial differences [34]. The cranial base angle can potentially influence the sagittal position of the jaws and therefore go some way to explaining the differences in races.

The prevalence of Class III malocclusion in a Chinese population is higher than Caucasian population (3-4%), which has been noted to be around 13% and has even been as high as 23% [21,35,36]. It is important when comparing such studies to remember that differing criteria for subject selection and differing indices may have been used. A previous comparison between Chinese and Caucasian Class III surgical cases found a difference in linear cranial base morphology, but not angular cranial base morphology [37]. A significant linear difference was noted in both anterior and posterior cranial bases, where the Chinese sample had a shorter anterior but longer posterior cranial base. Unfortunately, the sample size in that study was on the small side, with only 30 subjects overall (Caucasian = 14, Chinese = 16).

Our study had a sample size of 83 subjects with three different sagittal skeletal patterns and a more homogeneous original of southern China; it expanded the dataset for the cranial base-jaw base relationship in a Chinese population. The results reinforce the previous study of Chinese surgical Class III cases [37]. Class III is known to have a strong genetic element and in this study it was found that the correlation between cranial base and jaw base was closer in skeletal Class III cases.

In the future population study, a bigger sample size can be considered. Besides, 3-dimensional cone beam computer tomography (CBCT) is more viable than two dimension cephalometric radiographs and can also solve the problem of image overlapping. Further investigation can be carried out in the future to evaluate the relationship between cranial base and jaw base 3-dimensionally for a specific population.

## Conclusions

This study looked into a southern Chinese population to investigate a possible link between sagittal jaw relationships and the cranial base angle. It is found that the SNB angle decreases as the cranial base angle increases; the short NBa is, the shorter the maxillary length is. The cranial base appeares to have a certain correlation with the jaw base relationship in a southern Chinese population, and the correlation tends to be closer in skeletal Class III cases.

## Competing interests

The authors declare that they have no conflict of interests.

## Authors’ contributions

YY designed the study and calibrated the investigator who made the cephalogram measurements. AC measured the cephalograms and analyzed the data. SP analyzed and interpreted the data and drafted the manuscript. CL drafted the manuscript and drew the figure. All of the authors read and approved the final manuscript.
